# Microradiography of Microcalcifications in Breast Specimen: A New Histological Correlation Procedure and the Effect of Improved Resolution on Diagnostic Validity

**DOI:** 10.1155/2012/526293

**Published:** 2012-10-11

**Authors:** H.-J. Langen, S. Koehler, J. Bielmeier, R. Jocher, D. Kranzfelder, N. Jagusch, G. Treutlein, Th. Wetzler, J. Müller, G. Ott

**Affiliations:** ^1^Department of Radiology, The Medical Mission Clinic, Salvatorstra**β**e 7, 97074 Wuerzburg, Germany; ^2^Department of Gynecology, The Medical Mission Clinic, Salvatorstra**β**e 7, 97074 Wuerzburg, Germany; ^3^Private Practice, Center for Radiological Diagnostics, Eichhornstra**β**e 21, 97070 Wuerzburg, Germany; ^4^Department of Pathology, the University of Wuerzburg, 97080 Wuerzburg, Germany

## Abstract

*Introduction*. Does high-resolution visualization of microcalcifications improve diagnostic reliability? *Method*. X-rays were taken of mamma specimens with microcalcifications in 32 patients (10 malignant; 22 benign) using conventional radiography (12 Lp/mm) and high-resolution radiography (2000 Lp/mm). Histological sections were subsequently prepared and correlated to the microradiographic image and every calcification was assigned an exact malignant or benign histological diagnosis. Five radiologists classified single groups of calcifications in both methods according to the BIRADS classification system. *Results*. Using microradiography microcalcifications can be shown in high resolution at the cell level including histological correlation. In some cases, the diagnostic validity was improved by the high resolution in microradiography. In other cases, the high resolution resulted in more visible calcifications, thus giving benign calcifications a malignant appearance. In the BIRADS 2 and 3 group, the probability of malignancy was 28.6% in the conventional radiography evaluation and 37.8% in the microradiography evaluation. In the BIRADS 4 and 5 group, the probability of malignancy was 34.2% in the conventional radiography evaluation and 24.4% in the microradiography evaluation. The differences were not significant. *Summary*. Overall, the improved resolution in microradiography did not show an improvement in diagnostic accuracy compared to conventional radiography.

## 1. Introduction

It is well known that benign and malignant changes of the breast show calcifications [1, 2]. Microcalcification analysis has been used to try to identify the histological process that formed the calcification and to determine the benign or malignant cause of the calcification. Although some calcification configurations have been clearly identified as benign or malignant, this is not possible for all calcifications [1–4]. Increased resolution has been used in an attempt to improve the diagnostic validity of microcalcification analysis [3]. The aim of this study is to correlate individual microcalcifications in breast specimens to an exact histological diagnosis using high resolution plates (2000 lines/mm) and to determine whether the particularly high resolution of microcalcifications provides improved diagnostic validity.

## 2. Materials and Methods

X-rays were retrospectively taken of paraffin embedded breast specimens from 32 patients. All specimens with a thickness of 3 mm contained microcalcifications (10 × malignant; 22 × benign; Tables 1 and 2). Conventional specimen radiography was performed using a conventional mammography device (Mammodiagnost 300, 25 kV, 19.8 mAs, focus film distance 65 cm, focus size 0.3 mm; Philips), film-screen radiography (Film Agfa Mamoray HDR-C Plus PQ; 12 lines/mm). High-resolution specimen radiography was also performed on all specimens (Kodak high-resolution plates Type 1A; 2000 lines/mm) using a special device for specimen radiography (43855A Faxitron X-ray, Wheeling, IL, USA). The exposure time was 6 hours at 20 kV, 2.5 mA, 30 cm focus film distance, focus 0.5 mm. Histological cuts (hematoxylin-eosin staining) were made from the specimens and calcifications on the microradiographic picture were correlated to the corresponding histological cut. In cases in which the calcifications in the histological cut were largely washed out, the correlation to a histological region was made by the shape of the specimen. Every calcification was assigned an exact histological diagnosis in this manner. This procedure prevents benign calcifications in the vicinity of malignant tumors from being classified as malignant. The microradiographic images and histological specimens were documented digitally using a microscope. Five radiologists with mammography experience classified single groups of calcifications on conventional mammography according to the BIRADS classification (BIRADS 2–5) [5]. The digitalized high-resolution films were then evaluated on a monitor in a random order (Table 1). The single groups of calcifications were rotated and mirrored with respect to the conventional film in order to prevent memory of the conventional film from influencing the results. The groups of BIRADS 2 and 3 and the groups of BIRADS 4 and 5 were combined to form one group (Table 2). These were then evaluated with respect to the risk of malignancy. The differences between conventional mammography and microradiography were checked using the chi-square test. The differences were considered statistically significant at a significance level of *P* < 0.05.

## 3. Results

**Figure 1:**
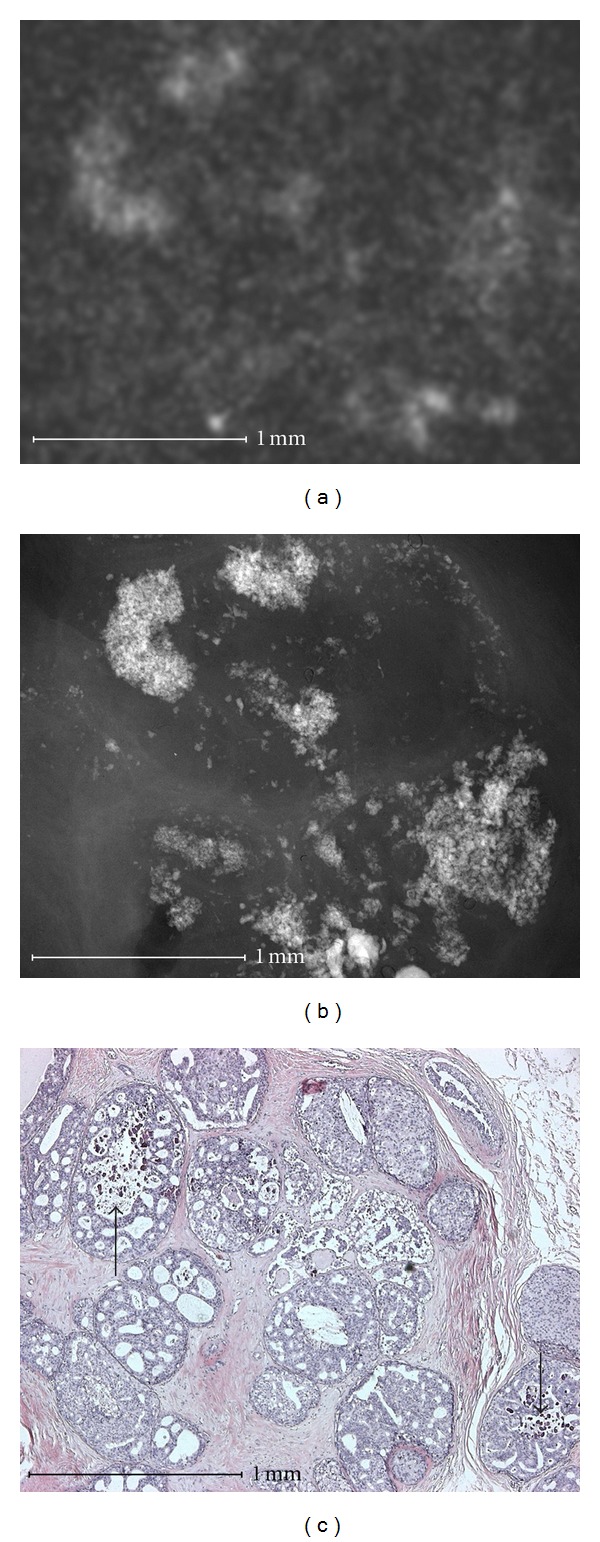
Cribriform intraductal carcinoma in situ (nonhigh grade). Microcalcifications (b) that are amorphous and indistinct in microradiography and in conventional specimen radiography (a). Histological evidence of gravel-like calcifications (arrow in (c)). In the histological as well as the microradiographic image, the calcifications have not yet condensed to form larger structures compared to Figure 2.

**Figure 2:**
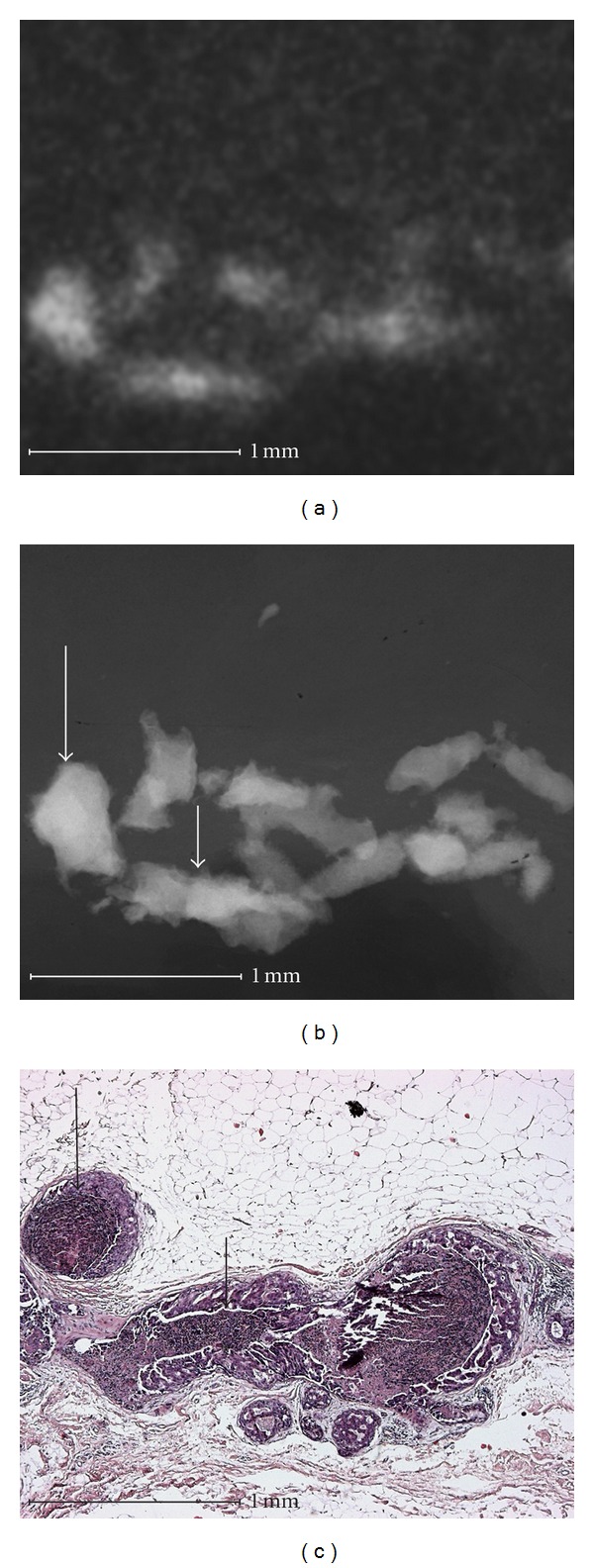
Intraductal comedo type breast carcinoma (high grade). Microradiography shows fine linear calcifications with smooth borders (large arrow in (b)) in which the calcified tumor necrosis completely fills the duct (large arrow in (c)). Linear calcifications with rough, irregular borders are also shown (small arrow in (b)). In these areas the calcifications are not significantly progressed and they do not completely fill the duct (small arrow in (c)). These microradiographic differences are not detectable in conventional specimen radiography (a).

**Figure 3:**
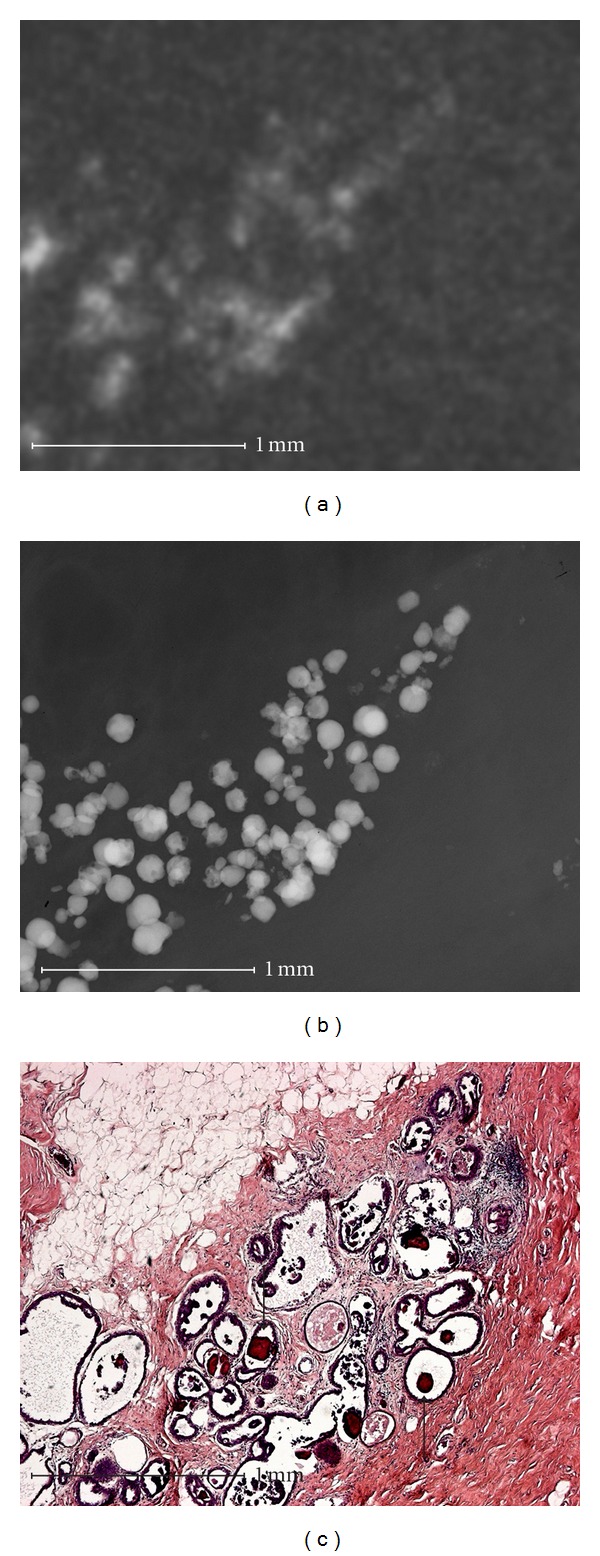
Fibrocystic mastopathy with sclerosing adenosis. Round microcalcification that is typically benign in microradiography (b) appears linear and amorphous in conventional specimen radiography as a result of the superposition (a). The calcification is marked by arrows in the histological picture (c).

**Figure 4:**
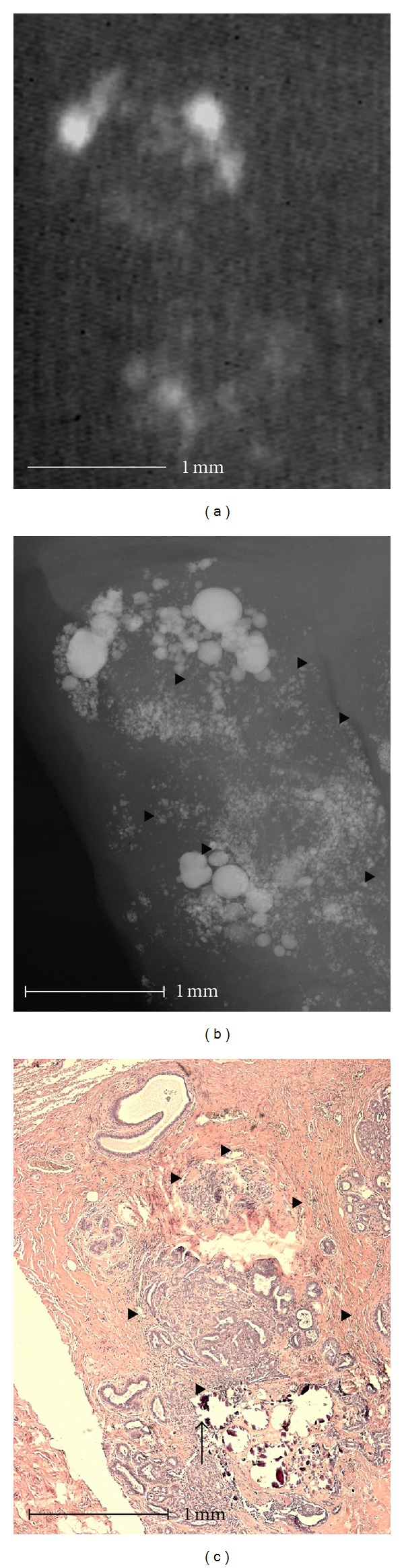
Fibrocystic mastopathy with sclerosing adenosis. Conventional specimen radiography show some amorphous and indistinct calcifications (a). The amorphous calcifications are microradiographically displayed as a summation effect of round and smooth calcifications of varying sizes. Diffuse amorphous microcalcifications are also visible (triangles in (b)) and result in the spotted shadowing in conventional specimen radiography. Fibrocystic mastopathy with sclerosing adenosis is demonstrated histologically (c). Only a few fragments of the large round calcifications in the cysts are histologically visible (arrow in (c)). The amorphous calcifications are almost completely lost during histological preparation. As a result of the shape of the calcification area and the specimen, the amorphous calcifications can be clearly correlated to a histological area of sclerosing adenosis (triangles in (b) and (c)). The calcifications are intraluminal calcifications in sclerosing adenosis.

Using microradiography microcalcifications can be shown in high resolution at the cell level including a histological correlation (Figures 1–4).

Amorphous and indistinct microcalcifications (Figure 1) were able to be correlated in one case to a ductal cribriform carcinoma. The calcifications developed in dead water spaces between tumor cells and were not condensed to form a tubular structure. The correlation of faint shadows to anatomical structures is difficult in conventional specimen radiography.

Analysis of individual cases showed that the high resolution of microradiography improved, worsened, or did not change the evaluation of calcifications with respect to malignancy.

### 3.1. Examples of Unchanged Diagnostic Validity by Microradiography

Linear calcifications with smooth and indistinct borders (Figure 2) can be correlated histologically to an intraductal calcification in tumor necrosis, which is surrounded by intraductally growing tumor tissue. In linear calcifications with smooth borders the calcified tumor necrosis completely filled out the duct and was only surrounded by a thin layer of vital tumor cells. In addition, linear calcification with rough borders can be identified. In these areas the calcified tumor necrosis is not in an advanced stage and does not completely fill out the duct, and the surrounding layer of vital tumor cells is clearly thicker. These microradiographic differences cannot be recognized using conventional specimen radiography. But this effect did not influence the diagnostic validity between conventional radiography and microradiography. The calcifications in Figure 2 were assigned to BIRADS 4 and 5 by four examiners with both methods (Table 2, Case 31).

### 3.2. Examples of Improved Diagnostic Validity by Microradiography

Groups of round calcifications (Figure 3) can be recognized histologically as fibrocystic mastopathy with round psammoma body-like calcifications in dilated lobuli. The superposition in conventional specimen radiography causes the round calcifications to appear linear and amorphous. This effect leads to a different evaluation of the calcifications in microradiography than in conventional specimen radiography. Therefore, all five examiners assigned this group of round calcifications in microradiography to BIRADS 2 or 3, while all five examiners classified the specimens in images with conventional resolution as BIRADS 4 or 5 (Table 2, Case 27).

### 3.3. Examples of Inferior Diagnostic Validity by Microradiography

Almost identical amorphous and indistinct calcifications with a malignant cause in Figure 1 are also seen in Figure 4. In this case, the calcifications are caused by fibrocystic mastopathy with sclerosing adenosis. It is impressive that the indistinct calcifications that are shown via microradiography in Figure 4 are visible in conventional specimen radiography only as a faint shadow. The indistinct calcifications were almost completely eliminated during the histological procedure. Only a few fragments of the large round calcifications in the cystically dilated lobuli were visible histologically.

**Figure 5:**
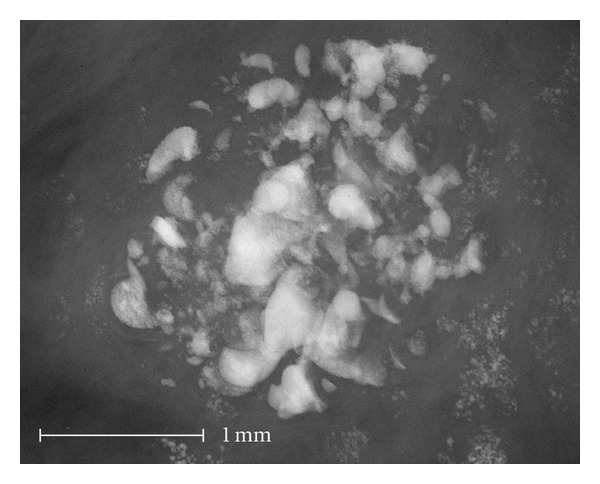
Fibrocystic mastopathy and adenosis. Microradiography demonstrates fine pleomorphic calcifications. This implies a malignant genesis as the cause of calcification. However, histology only detects adenosis in which the dilated ducts and acini are deformed that tracing of the irregular wall borders shows fine pleomorphic calcifications.

These benign calcifications showed additional small calcifications in high resolution (Figure 4) or more irregular borders (Figure 5). Therefore, these benign calcifications appear amorphous in conventional radiography and were classified as BIRADS 2 and 3 by 5 examiners in Figure 4 (Table 2, Case 10) and 3 examiners in Figure 5, respectively, (Table 2, Case 16). When using the high resolution images, the calcifications were classified as BIRADS 4 and 5 by 4 examiners in Figure 4 (5 examiners in Figure 5, resp.).

Although the diagnostic validity due to the higher resolution in microradiography is improved in single cases (Figure 3), overall, the higher resolution did not provide better diagnostic validity than that of conventional specimen radiography (Figures 4 and 5). The diagnostic validity for microradiography with respect to the probability of malignancy was worse than that of conventional specimen radiography (Table 3).

However, the differences were not significant. In the BIRADS 2 and 3 group (Table 2) the probability of malignancy was 24 of 84 (28.6%) in the conventional radiography evaluation and 31 of 82 (37.8%) in the microradiography evaluation. The differences were not significant with a *P* value of 0.18. In the BIRADS 4 and 5 group the probability of malignancy was 26 of 76 (34.2%) in the conventional radiography evaluation and 19 of 78 (24.4%) in the microradiography evaluation. These differences were also not significant with a *P* value of 0.16.

## 4. Discussion

When detecting breast carcinomas via mammography, the evidence of calcification in addition to soft tissue lesions plays an important role. The most important components of the assessment of microcalcifications are the morphology of the individual calcifications and the configuration of the group [6]. The calcification morphology is the most important and independent parameter of the assessment of a cluster of microcalcifications [6]. To determine a benign or malignant histological diagnosis on the basis of the shape of a calcification, it is useful to understand the histological process that caused the calcification [4]. Although a number of papers have examined the use of the magnification technique for improving the visibility of microcalcifications [1–3], there are only a few reports that analyze the shape of microcalcifications. Lanyi [7] addressed this problem by analyzing mammograms and specimen radiographies via a magnifying glass. The poor resolution was compensated for by recording the calcifications and producing magnifications on paper. The drawings and films were correlated to the histological cuts. Lanyi discovered that the calcifications in the case of adenosis could be flat or facetted on one or more sides due to the pressure of one or more adjacent calcifications or corresponding cysts. In our study, however, the calcifications in the case of adenosis appeared very irregular in the individual cases of high resolution specimen radiography. These findings are much more pronounced than expected according to the results of Lanyi or our own conventional specimen radiography. In these cases, the high resolution resulted in benign calcifications appearing malignant. In the case of intraductal carcinomas, Lanyi discovered with this method [7] that calcification starts centrally in tumors and typically in the shape of a dot or bean. In more progressed tumors, the calcifications condensed into linear shapes, while the occurrence of dot and bean-shaped calcifications decreased. We were also able to show this type of calcification in intraductal carcinomas with some of the linear calcifications having a smooth border and some having a rough and irregular border in microradiography. We traced the irregular calcification delineation to a thick layer of surrounding vital intraductal tumor tissue, while the calcifications with smooth borders were only surrounded by a thin layer of intraductal tumor tissue. For the cribriform intraductal carcinoma, Lanyi [7] showed that the sponge-like structure of the tumor resulted in the development of spaces which are ideal for the precipitation of calcium. These sieve-like spaces are filled with round calcifications, so the round calcifications are predominant in this tumor type. Amorphous and indistinct calcifications which have not yet condensed to form round calcifications were seen in our cribriform carcinoma case. In general, microradiography provides significantly better visualization of microcalcifications than the method of Lanyi. This allows an optimal structural analysis of microcalcifications as well as an exact histological correlation to the cell level.

The histopathological reason for different types of calcifications can be demonstrated effectively. The comparison of the structural analysis of microcalcifications in conventional specimen radiography and microradiography shows that the typical benign calcifications in microradiography may appear malignant in conventional radiography due to superposition. In opposition to our expectations, this study did not show an improvement in diagnostic accuracy when evaluating microcalcifications using microradiography compared to conventional radiography. An improvement in diagnostic validity was only shown in a few cases with benign microcalcifications, but this was offset by the irregular visualization of the benign microcalcifications caused by the higher resolution, resulting in a higher BIRADS category. The diagnostic validity for microradiography with respect to the probability of malignancy tended to be worse than that of conventional specimen radiography. Similar results were demonstrated by Grunert et al. [1] when determining tumor extension on the basis of microcalcification in specimen radiography. Using a magnification factor of 4, the tumor borders were clearly overestimated compared to an examination using a magnification factor of only 1.5. With a constant sensitivity, specimen radiography using a magnification factor of 4 results in significantly worse specificity for determining tumor borders. The examiners probably need to first become familiar with the appearance of microcalcifications in magnification radiography in order to achieve an improvement in diagnostic validity. Therefore, higher resolution with improved presentation of microcalcifications by itself is not sufficient for improving diagnostic validity.

In our study, the probability for malignancy in the BIRADS 2 and 3 group was very high at more than 20%. Normally BIRADS category 3 (“probably benign”) is associated with an estimated low risk of malignancy (<2%) [8]. The high risk in our study was probably caused by the evaluation of calcifications which led to tissue excision of the breast. The limited sample size may be another reason.

It is known that 13.6% of calcifications from breast specimens are lost during embedding and 12.6% are lost after embedding during cutting [9]. In addition, microcalcifications are washed out of specimens [6] during storage and fixation in water solutions, for example, formaldehyde, as was also observed in our study. The loss of calcifications in the histological specimen compared to in microradiography is demonstrated impressively with the described method.

## 5. Conclusion

Microradiography allows an exact structural analysis of microcalcifications with high accuracy and histological correlation. In some cases, the knowledge of the microradiographic appearance of breast microcalcifications improves the understanding of calcifications in mammography because they are the result of the superposition of microradiographic images. The improved resolution in mammography does not necessarily result in correct evaluation of microcalcifications. An improvement can probably be achieved by examiners becoming familiar with high-resolution radiography. For future studies, microradiography can help determine the degree to which higher resolution is useful in mammography, even though the procedure can only be used on specimens.

Since many microcalcifications can not be assigned surely to a benign or malignant cause, when in doubt the diagnosis must be confirmed by biopsy.

## Figures and Tables

**Table:** (a) Results for examiner 1

Examiner 1																	
	Conventional specimen radiography						Microradiography										
	BIRADS				Correct/false		BIRADS				Correct/false		Histology		Change by microradiography		
	2	3	4	5			2	3	4	5			Benign/malignant				
Case 1			×			False		×			Correct		Benign		Correct		
Case 2				×		False			×			False	Benign			No (f)	
Case 3		×			Correct			×			Correct		Benign			No (f)	
Case 4		×			Correct				×			False	Benign				False
Case 5		×			Correct			×			Correct		Benign			No (c)	
Case 6			×			False			×			False	Benign			No (f)	
Case 7		×			Correct				×			False	Benign				False
Case 8		×			Correct			×			Correct		Benign			No (c)	
Case 9			×			False			×			False	Benign			No (f)	
Case 10		×			Correct				×			False	Benign				False
Case 11		×			Correct				×			False	Benign				False
Case 12			×			False			×			False	Benign			No (f)	
Case 13	×					False		×				False		Malignant		No (f)	
Case 14				×	Correct				×		Correct			Malignant		No (c)	
Case 15	×					False	×					False		Malignant		No (f)	
Case 16			×			False			×			False	Benign			No (f)	
Case 17		×			Correct			×			Correct		Benign			No (c)	
Case 18				×		False			×			False	Benign			No (f)	
Case 19		×			Correct		×				Correct		Benign			No (c)	
Case 20		×			Correct			×			Correct		Benign			No (c)	
Case 21		×				False		×				False		Malignant		No (f)	
Case 22			×		Correct			×				False		Malignant			False
Case 23			×		Correct				×		Correct			Malignant		No (c)	
Case 24			×			False			×			False	Benign			No (f)	
Case 25			×		Correct			×				False		Malignant			False
Case 26		×			Correct			×			Correct		Benign			No (c)	
Case 27			×			False		×			Correct		Benign		Correct		
Case 28			×			False				×		False	Benign			No (f)	
Case 29		×			Correct				×			False	Benign				False
Case 30		×				False		×				False		Malignant		No (f)	
Case 31			×		Correct				×		Correct			Malignant		No (c)	
Case 32			×		Correct				×		Correct			Malignant		No (c)	

Total	2	14	13	3	18	14	2	13	16	1	13	19	22	10	2	10 (c)/13 (f)	7

**Table:** (b) Results for examiner 2

Examiner 2																	
	Conventional specimen radiography						Microradiography										
	BIRADS				Correct/false		BIRADS				Correct/false		Histology		Change by microradiography		
	2	3	4	5			2	3	4	5			Benign/malignant				
Case 1		×			Correct		×				Correct		Benign			No (c)	
Case 2		×			Correct		×				Correct		Benign			No (c)	
Case 3	×				Correct			×			Correct		Benign			No (c)	
Case 4		×			Correct					×		False	Benign				False
Case 5		×			Correct				×			False	Benign				False
Case 6			×			False			×			False	Benign			No (f)	
Case 7		×			Correct			×			Correct		Benign			No (c)	
Case 8	×				Correct					×		False	Benign				False
Case 9				×		False			×			False	Benign			No (f)	
Case 10		×			Correct			×			Correct		Benign			No (c)	
Case 11		×			Correct			×			Correct		Benign			No (c)	
Case 12			×			False			×			False	Benign			No (f)	
Case 13	×					False	×					False		Malignant		No (f)	
Case 14				×	Correct			×				False		Malignant			False
Case 15	×					False	×					False		Malignant		No (f)	
Case 16		×			Correct				×			False	Benign				False
Case 17		×			Correct		×				Correct		Benign			No (c)	
Case 18				×		False			×			False	Benign			No (f)	
Case 19	×				Correct			×			Correct		Benign			No (c)	
Case 20		×			Correct			×			Correct		Benign			No (c)	
Case 21	×					False	×					False		Malignant		No (f)	
Case 22				×	Correct				×		Correct			Malignant		No (c)	
Case 23				×	Correct			×				False		Malignant			False
Case 24		×			Correct					×		False	Benign				False
Case 25		×				False		×				False		Malignant		No (f)	
Case 26			×			False	×				Correct		Benign		Correct		
Case 27			×			False	×				Correct		Benign		Correct		
Case 28			×			False			×			False	Benign			No (f)	
Case 29			×			False				×		False	Benign			No (f)	
Case 30		×				False	×					False		Malignant		No (f)	
Case 31		×				False			×		Correct			Malignant	Correct		
Case 32			×		Correct				×		Correct			Malignant		No (c)	

Total	6	14	7	5	18	14	9	9	10	4	14	18	22	10	3	11 (c)/11 (f)	7

**Table:** (c) Results for examiner 3

Examiner 3																	
	Conventional specimen radiography						Microradiography										
	BIRADS				Correct/false		BIRADS				Correct/false		Histology		Change by microradiography		
	2	3	4	5			2	3	4	5			Benign/malignant				
Case 1			×			False			×			False	Benign			No (f)	
Case 2			×			False			×			False	Benign			No (f)	
Case 3	×				Correct		×				Correct		Benign			No (c)	
Case 4	×				Correct				×			False	Benign				False
Case 5	×				Correct			×			Correct		Benign			No (c)	
Case 6		×			Correct				×			False	Benign				False
Case 7	×				Correct					×		False	Benign				False
Case 8			×			False				×		False	Benign			No (f)	
Case 9			×			False		×			Correct		Benign		Correct		
Case 10	×				Correct				×			False	Benign				False
Case 11		×			Correct				×			False	Benign				False
Case 12			×			False				×		False	Benign			No (f)	
Case 13	×					False		×				False		Malignant		No (f)	
Case 14				×	Correct				×		Correct			Malignant		No (c)	
Case 15	×					False	×					False		Malignant		No (f)	
Case 16		×			Correct					×		False	Benign				False
Case 17	×				Correct			×			Correct		Benign			No (c)	
Case 18			×			False			×			False	Benign			No (f)	
Case 19	×				Correct		×				Correct		Benign			No (c)	
Case 20			×			False	×				Correct		Benign		Correct		
Case 21		×				False	×					False		Malignant		No (f)	
Case 22				×	Correct					×	Correct			Malignant		No (c)	
Case 23				×	Correct					×	Correct			Malignant		No (c)	
Case 24		×			Correct			×			Correct		Benign			No (c)	
Case 25		×				False			×		Correct			Malignant	Correct		
Case 26	×				Correct			×			Correct		Benign			No (c)	
Case 27			×			False	×				Correct		Benign		Correct		
Case 28				×		False			×			False	Benign			No (f)	
Case 29	×				Correct					×		False	Benign				False
Case 30		×				False	×					False		Malignant		No (f)	
Case 31			×		Correct				×		Correct			Malignant		No (c)	
Case 32	×					False				×	Correct			Malignant	Correct		

Total	12	7	9	4	17	15	7	6	11	8	15	17	22	10	5	10 (c)/10 (f)	7

**Table:** (d) Results for examiner 4

Examiner 4																	
	Conventional specimen radiography						Microradiography										
	BIRADS				Correct/false		BIRADS				Correct/false		Histology		Change by microradiography		
	2	3	4	5			2	3	4	5			Benign/malignant				
Case 1			×			False			×			False	Benign			No (f)	
Case 2			×			False				×		False	Benign			No (f)	
Case 3	×				Correct			×			Correct		Benign			No (c)	
Case 4	×				Correct				×			False	Benign				False
Case 5	×				Correct			×			Correct		Benign			No (c)	
Case 6		×			Correct				×			False	Benign				False
Case 7	×				Correct				×			False	Benign				False
Case 8			×			False				×		False	Benign			No (f)	
Case 9				×		False		×			Correct		Benign		Correct		
Case 10	×				Correct				×			False	Benign				False
Case 11	×				Correct				×			False	Benign				False
Case 12		×			Correct					×		False	Benign				False
Case 13	×					False	×					False		Malignant		No (f)	
Case 14				×	Correct				×		Correct			Malignant		No (c)	
Case 15	×					False	×					False		Malignant		No (f)	
Case 16		×			Correct					×		False	Benign				False
Case 17	×				Correct		×				Correct		Benign			No (c)	
Case 18		×			Correct				×			False	Benign				False
Case 19	×				Correct		×				Correct		Benign			No (c)	
Case 20			×			False	×				Correct		Benign		Correct		
Case 21		×				False	×					False		Malignant		No (f)	
Case 22				×	Correct				×		Correct			Malignant		No (c)	
Case 23				×	Correct					×	Correct			Malignant		No (c)	
Case 24	×				Correct				×			False	Benign				False
Case 25			×		Correct			×				False		Malignant			False
Case 26		×			Correct			×			Correct		Benign			No (c)	
Case 27			×			False	×				Correct		Benign		Correct		
Case 28				×		False				×		False	Benign			No (f)	
Case 29	×				Correct					×		False	Benign				False
Case 30			×		Correct		×					False		Malignant			False
Case 31			×		Correct				×		Correct			Malignant		No(c)	
Case 32	×					False				×	Correct			Malignant	Correct		

Total	13	6	8	5	21	11	8	5	11	8	13	19	22	10	4	9 (c)/7 (f)	12

**Table:** (e) Results for examiner 5

Examiner 5																	
	Conventional specimen radiography						Microradiography										
	BIRADS				Correct/false		BIRADS				Correct/false		Histology		Change by microradiography		
	2	3	4	5			2	3	4	5			Benign/malignant				
Case 1				×		False		×			Correct		Benign		Correct		
Case 2				×		False				×		False	Benign			No (f)	
Case 3			×			False		×			Correct		Benign		Correct		
Case 4	×				Correct			×			Correct		Benign			No (c)	
Case 5		×			Correct				×			False	Benign				False
Case 6				×		False		×			Correct		Benign		Correct		
Case 7				×		False		×			Correct		Benign		Correct		
Case 8				×		False		×			Correct		Benign		Correct		
Case 9			×			False			×			False	Benign			No (f)	
Case 10		×			Correct				×			False	Benign				False
Case 11				×		False			×			False	Benign			No (f)	
Case 12			×			False		×			Correct		Benign		Correct		
Case 13			×		Correct		×					False		Malignant			False
Case 14				×	Correct				×		Correct			Malignant		No (c)	
Case 15	×					False	×					False		Malignant		No (f)	
Case 16				×		False			×			False	Benign			No (f)	
Case 17		×			Correct			×			Correct		Benign			No (c)	
Case 18			×			False				×		False	Benign			No (f)	
Case 19		×			Correct		×				Correct		Benign			No (c)	
Case 20			×			False	×				Correct		Benign		Correct		
Case 21		×				False	×					False		Malignant		No (f)	
Case 22				×	Correct		×					False		Malignant			False
Case 23				×	Correct			×				False		Malignant			False
Case 24				×		False		×			Correct		Benign		Correct		
Case 25	×					False	×					False		Malignant		No (f)	
Case 26		×			Correct		×				Correct		Benign			No (c)	
Case 27			×			False	×				Correct		Benign		Correct		
Case 28				×		False				×		False	Benign			No (f)	
Case 29		×			Correct		×				Correct		Benign			No (c)	
Case 30		×				False		×				False		Malignant		No (f)	
Case 31			×		Correct		×					False		Malignant			False
Case 32				×	Correct		×					False		Malignant			False

Total	3	8	8	13	13	19	12	11	6	3	15	17	22	10	9	6 (c)/10 (f)	7

**Table 2:** BIRADS classification in conventional specimen radiography and microradiography. Summary of five examiners.

Case	Numbers of diagnosis conv. radiography		Numbers of diagnosis microradiography		Histology		Diagnosis of the examined specimen
Nr.	BIRADS 2 and 3 (benign)	BIRADS 4 and 5 (malignant)	BIRADS 2 and 3 (benign)	BIRADS 4 and 5 (malignant)		
1	1	4	3	2	Benign		Fibrocystic mastopathy
2	0	5	1	4	Benign		Fibrocystic mastopathy
3	4	1	5	0	Benign		Fibrocystic mastopathy
4	5	0	1	4	Benign		Fibrocystic mastopathy
5	5	0	3	2	Benign		Fibrocystic mastopathy
6	2	3	1	4	Benign		Fibrocystic mastopathy
7	4	1	2	3	Benign		Fibrocystic mastopathy
8	2	3	2	3	Benign		Fibrocystic mastopathy
9	0	5	2	3	Benign		Fibrocystic mastopathy
10	5	0	1	4	Benign		Fibrocystic mastopathy
11	4	1	1	4	Benign		Fibrocystic mastopathy
12	1	4	1	4	Benign		Fibrocystic mastopathy
13	4	1	5	0		Malignant	Intraductal high-grade comedocarcinoma
14	0	5	1	4		Malignant	Ductal carcinoma in situ
15	5	0	5	0		Malignant	Ductal carcinoma in situ
16	3	2	0	5	Benign		Fibrocystic mastopathy
17	5	0	5	0	Benign		Fibrocystic mastopathy
18	1	4	0	5	Benign		Fibrocystic mastopathy
19	5	0	5	0	Benign		Fat tissue necrosis
20	2	3	5	0	Benign		Pericanalicular fibroadenoma
21	5	0	5	0		Malignant	Ductal carcinoma in situ
22	0	5	2	3		Malignant	Ductal carcinoma in situ
23	0	5	2	3		Malignant	Ductal carcinoma in situ
24	3	2	2	3	Benign		Fibrocystic mastopathy
25	3	2	4	1		Malignant	Ductal carcinoma in situ
26	4	1	5	0	Benign		Fibrocystic mastopathy
27	0	5	5	0	Benign		Fibrocystic mastopathy
28	0	5	0	5	Benign		Fibrocystic mastopathy
29	4	1	1	4	Benign		Fibrocystic mastopathy
30	4	1	5	0		Malignant	Intraductal high-grade comedocarcinoma
31	1	4	1	4		Malignant	Intraductal high-grade comedocarcinoma
32	2	3	1	4		Malignant	Cribriform intraductal carcinoma in situ
correct	60	26	51	19			
false	24	50	31	59			

Total	84	76	82	78			

**Table 3:** Change of diagnosis by microradiography.

	Correct change	None change		False change	Total
	Correct diagnosis in microradiography	Correct diagnosis in conventional and microradiography	Incorrect diagnosis in conventional and microradiography	Incorrect diagnosis in microradiography
Examiner 1	2	10	13	7	32
Examiner 2	3	11	11	7	32
Examiner 3	5	10	10	7	32
Examiner 4	4	9	7	12	32
Examiner 5	9	6	10	7	32

Total	23	46	51	40	160
